# 

**Published:** 1873-04

**Authors:** 


The Association of American Medical Editors.—The Association of American Medical Editors meets at St. Louis. on the evening of May 5th. The meeting will be held in the Polytechnic Building, corner of Chestnut and 7th streets, and will commence promptly at 8 o'clock P. M. It is hoped that this meeting will be largely attended, and all editors and assistant editors of regular Medical Journals are invited to connect themselves with the Association.

### Books and Pamphlets Received.

Proceedings of the Royal Society, Nos. 135-139. London: Taylor & Francis, 1872. Received through Wm. Wesley, Agent, Smithsonian Institution, London.

Dental Caries and its Causes. An investigation into the Influences of Fungi in the destruction of the Teeth. By Drs. Leber and Rottenstein. Translated by Thos. H. Chandler, D. M. D. With illustrations. Philadelphia: Lindsay & Blakiston, 1873. Buffalo: T. Butler & Son.

Fistula, Hæmorrhoids, Painful Ulcer, Stricture, Prolapsus, and other diseases of the Rectum, their Diagnosis and Treatment. William Allingham, F. R. C. S., etc. Second Edition. Philadelphia: Lindsay & Blakiston, 1873 Buffalo: T. Butler & Son.

A Hand-Book of Post Mortem Examinations and of Morbid Anatomy. By Francis Delafield, M. D. New York: Wm. Wood & Co., 1872. Buffalo: H. H. Otis.

Diseases of the Urinary Organs, including Strictures of the Urethra, Affections of the Prostate, and Stone in the Bladder. By John W. S. Gouley, M D., etc. New York: Wm. Wood &c., 1873. Buffalo: H. H. Otis.

Abnormal Reaction of the Acoustic Nerve in Chlorosis and Bright's Disease. By Wm. B. Neftel, M. D. Reprinted from the Archives of Scientific and Practical Medicine. Jan. 1873.

Intussusception. By Stephen Rogers, M. D. New York: Reprinted from the Transactions of the New York State Medical Society for 1872.

Reports of the Superintendent and Trustees of the Butler Hospital for the Insane. Jan. 1873.

Nineteenth Annual Report of the Trustees of the State Lunatic Asylum at Taunton, Mass.

Manual of Chemical Analysis as applied to the examination of Medicinal Chemicals. A Guide for the Determination of their Identity and Quality, and for the Detection of Impurities and Adulterations. By Frederick Hoffmann, Ph. D., etc. New York: D. Appleton & Co., 1873. Buffalo: Martin Taylor.

Catalogue of the Officers and Alumni of the Bellevue Hospital Medical College, 1861–1871. New York: D. Appleton & Co., 1873.

Half Hour Recreations in Popular Science. Dana Estes, Editor; No. 7. The Geology of the Stars. By Prof. A. Winchell. Boston: Estes & Lauriat, 1873. Buffalo: Martin Taylor.

Florida and South Carolina as Health Resorts. Read before the Massachusetts Medical Society, June, 1872. By W. W. Morland, M. D. Harv.

Quarterly Summary of the Transactions of the College of Physicians of Philadelphia. Collins, Printer, 1873.

Notes on a New Form of Percolator, the Triplex Pill, Rhubarb, Aconite Root, etc., etc. By Edward R. Suibb, M. D.

Twenty-Fourth Annual Report of the Managers of The Western House of Refuge of the State of New York.

Contributions to the Diagnosis of Cancerous Diathesis—Carcinosis. By Wm. B. Neftel, M. D. Re-printed from The Archives of Scientific and Practical Medicine.

Report of the Resident Physician of Brigham Hall, a Hospital for the Insane. For the year 1872.

The Treatment of Whooping-Cough with Quinine. By B. F. Dawson, M. D. Re-printed from American Journal of Obstetrics and Diseases of Women and Children. Vol. V., No. IV. New York: Wm. Woods & Co.

The Logic of Medicine. An Address Delivered on the Occasion of the Twenty-fifth Anniversary of the New York Academy of Medicine. By Edward S. Dunster, M. D. New York: D. Appleton & Co. Re-print from New York Medical Journal, Mar., 1873.

Fourth Annual Report of the Trustees of the Willard Asylum for the Insane. For 1872.

Ophthalmic Contributions. I. Dermoid Tumor of the Cornea. II. An Additional Method for the Determination of Astigmatism. III. Cyst of the Iris, Removed by Operation. By George Strawbridge, M. D. Philadelphia: Lindsay & Blakiston, 1873.

Proceedings of the Third Meeting of the American Association for the Cure of Inebriates. Held in New York, Oct. 8th, 9th, and 10th, 1872.

Report on the Progress of Ophthalmology, 1872. Prepared for the American Ophthalmological Society. By B. Joy Jeffries, A. M., M. D.

The True Object of Medical Legislation. By Stephen Rogers, M. D.

A New Method of Treating Strictures of the Urethra after External Sections. By C. H. Masten, M. D., Mobile, Ala.

The Criminal Use of Proprietary or Advertised Nostrums. By Ely Van De Warker, M. D., Syracuse, N. Y.

Annual Report of the Board of Health, to the General Assembly of Louisiana, Dec. 31, 1872.

THE BUFFALO

**Medical and Surgical Journal**

PUBLISHED MONTHLY,

Containing Original Articles, Reports of Medical Societies and Hospitals, Editorial, Reviews, Correspondence, Army News, etc., etc.; including the usual variety of Medical Periodical Publications. Specimen copies sent on application.

Address Buffalo Medical & Surgical Journal, Buffalo, N. Y.

TERMS OF SUBSCRIPTION, - - $3.00 PER YEAR, IN ADVANCE.

